# U mobilization and associated U isotope fractionation by sulfur-oxidizing bacteria

**DOI:** 10.3389/fmicb.2023.1190962

**Published:** 2023-07-18

**Authors:** C. D. Rosendahl, Y. Roebbert, A. Schippers, S. Weyer

**Affiliations:** ^1^Leibniz Universität Hannover, Institut für Mineralogie, Hannover, Germany; ^2^Federal Institute for Geosciences and Natural Resources (BGR), Geomicrobiology, Hannover, Germany

**Keywords:** uranium, isotope fractionation, remobilization, laboratory batch experiments, *Thiobacillus denitrificans*, *Acidithiobacillus ferrooxidans*

## Abstract

Uranium (U) contamination of the environment causes high risk to health, demanding for effective and sustainable remediation. Bioremediation via microbial reduction of soluble U(VI) is generating high fractions (>50%) of insoluble non-crystalline U(IV) which, however, might be remobilized by sulfur-oxidizing bacteria. In this study, the efficacy of *Acidithiobacillus (At.) ferrooxidans* and *Thiobacillus (T.) denitrificans* to mobilize non-crystalline U(IV) and associated U isotope fractionation were investigated. *At. ferrooxidans* mobilized between 74 and 91% U after 1 week, and U mobilization was observed for both, living and inactive cells. Contrary to previous observations, no mobilization by *T. denitrificans* could be observed. Uranium mobilization by *At. ferrooxidans* did not cause U isotope fractionation suggesting that U isotope ratio determination is unsuitable as a direct proxy for bacterial U remobilization. The similar mobilization capability of active and inactive *At. ferrooxidans* cells suggests that the mobilization is based on the reaction with the cell biomass. This study raises doubts about the long-term sustainability of *in-situ* bioremediation measures at U-contaminated sites, especially with regard to non-crystalline U(IV) being the main component of U bioremediation.

## Synopsis

Non-crystalline U(IV) mobilization with sulfur-oxidizing bacteria is studied to provide information about the long-term sustainability of *in-situ* bioremediation measures of U-contaminated sites.

## 1. Introduction

Uranium is a trace metal with a concentration of about 1.5 ppm in the continental crust, most of which is incorporated in accessory minerals, e.g., zircon, apatite, xenotime which are formed during late stage crystallization processes (Salters, 2018). Economically important uranium deposits mainly consist of uraninite and other refractory U-bearing minerals (Cuney, 2009). The ionizing radiation and the toxicity of U poses a considerable health risk to most life forms. A special risk of U poisoning is posed by the ingestion of U-contaminated drinking water, even with moderately increased U concentrations on a long-term scale of several years or decades, resulting in severe kidney damage and organ failure (Konietzka et al., 2005; Höller et al., 2009; Dienemann and Utermann, 2012). Due to its health hazard, effective *in-situ* remediation measures are necessary for U-contaminated sites.

Reasons for an environmental U contamination can be divers, e.g., mining and milling, military application, and illegal and inappropriate disposal (Dienemann and Utermann, 2012; Zammit et al., 2014). Oxidizing environments can form highly soluble uranyl compounds and U complexes (Abdelouas, 2006) which can be transported over long distances by water. Further U enrichment in the environment can emerge by a naturally elevated U content in bedrock and soils, application of U-containing mineral phosphor fertilizers and by groundwater intrusion into permanent repository sites for nuclear waste (Dienemann and Utermann, 2012).

Such contaminations require an effective *in-situ* treatment. Physical and chemical remediation measures are limited by their high cost and low sustainability (Lovley and Phillips, 1992). In contrast, bioremediation by (micro)biological reduction of soluble U(VI) to sparingly soluble U(IV), e.g., by *Shewanella (S.) oneidensis*, appears to be most promising (Finneran et al., 2002; Wall and Krumholz, 2006; Newsome et al., 2014, 2015; Lakaniemi et al., 2019). A major problem of bioremediation by bacteria is the formation of biomass-associated non-crystalline U(IV), which is assumed to be a mixture of compounds including coordination to carboxylic, phosphate, or silicate moieties (Wang et al., 2013; Alessi et al., 2014). The proportion of non-crystalline U(IV) formed by *S. oneidensis* MR-1 can amount from 50% to almost 100% depending on the presence of dissolved solutes, e.g., phosphate, silicate, sulfate, which naturally occur in sediments, soils or groundwater, and depending on the U concentration (Stylo et al., 2013). Non-crystalline U(IV) is considered to be more labile than uraninite. Its fast oxidation during oxygen exposure, by nitrate or by ligand complexation (Cerrato et al., 2013; Newsome et al., 2015; Roebbert et al., 2021) demands for more detailed investigations regarding its stability in the environment in order to assess the efficiency and vulnerability of bioremediation.

A wide range of microorganisms is generally able to aerobically and/or anaerobically oxidize U either indirectly, e.g., via Fe(III) or chelators, or directly via electron transfer from the bacterial cell surface to the electron acceptor, e.g., O_2_, Fe(III), nitrate (Guay et al., 1977; Soljanto and Tuovinen, 1980; Kalinowski et al., 2004). Several sulfur-oxidizing bacteria, which oxidize reduced sulfur compounds as electron donors for energy conservation, are also known to oxidize Fe(II) and even U(IV) under special environmental conditions such as low pH. Two of those bacteria, *At. ferrooxidans* and *T. denitrificans*, were chosen in this study in order to determine the remobilization potential of sulfur-oxidizing bacteria for non-crystalline U(IV). *At. ferrooxidans* is a facultative anaerobic, Gram-negative, obligate chemolithoautotrophic, extreme acidophilic bacterium (Quatrini and Johnson, 2019) with optimal growth conditions of 30–35°C and pH 2.5 (Schippers et al., 2014; Quatrini and Johnson, 2019). It has often been isolated from mining-impacted environments and has a major impact on the biogeochemical cycles in low-pH environments regarding Fe, S, H (Quatrini and Johnson, 2019; Hedrich and Schippers, 2021), and is involved in the solubilization of U from ores (Bosecker and Wirth, 1980). DiSpirito and Tuovinen (1981, 1982) described *At. ferrooxidans’* ability to directly oxidize U(IV), supplied as uranous sulfate or uranous oxide, coupled with carbon dioxide fixation by the use of the conserved energy. *T. denitrificans* is a facultative anaerobic, Gram-negative obligate chemolithoautotrophic bacterium with an optimal pH around 7 and a temperature of around 30°C for growth (Kelly and Wood, 2000). The bacterium is found in various environments like soil, mud, freshwater and marine sediments as well as domestic sewage, industrial waste-treatment lagoons and digestion tanks (Kelly and Wood, 2000). The bacterium utilizes both oxygen and nitrate as electron acceptors. During anaerobic growth thiosulfate oxidation is coupled to nitrate reduction (Schedel and Trüper, 1980). Beller (2005) demonstrated that *T. denitrificans* is capable of anaerobic, nitrate-dependent oxidative dissolution of synthetic and biogenic U(IV) oxides, whereby nitrate-reduction was coupled to the presence of another electron donor like H_2_. In order to investigate the efficacy of the oxidation of non-crystalline U(IV) by sulfur-oxidizing bacteria, mobilization experiments with both, *At. ferrooxidans* and *T. denitrificans*, were implemented.

Furthermore, U isotope fractionation, associated with microbial U mobilization, was investigated as a potential monitor for the mobilization process. A range of U reduction experiments using sulfate or metal-reducing bacteria, e.g., *Geobacter sulfurreducens*, *Anaeromyxobacter dehalogenans*, *Shewanella* sp., *Desulfitobacterium* sp. and *Desulfovibrio brasiliensis,* showed significant U isotope fractionation (expressed as *ε* = 1,000‰ * (*α* − 1)) of 0.65‰ to 0.99‰ (Basu et al., 2014; Stirling et al., 2015; Stylo et al., 2015). This suggests that bacterial U(VI) reduction generally induces isotopic fractionation with ^238^U enrichment in the product U(IV) (Basu et al., 2014). Wang et al. (2015a) observed that oxidation of dissolved U(IV) with oxygen at acidic pH leads to isotopically lighter U(VI), while oxidation of solid U(IV) showed only a limited isotope effect. Recent research of Roebbert et al. (2021) showed isotope fractionation during the complexation of non-crystalline U(IV) by organic ligands leading to an enrichment of ^238^U in the mobilized fraction with δ^238^U = 0.2–0.7 ‰.

In this study, we conducted experiments with two species of bacteria, *At. ferrooxidans* and *T. denitrificans*, in order to determine the stability of non-crystalline U(IV) with respect to oxidation associated with sulfur-oxidizing bacteria. Furthermore, U isotope fractionation, associated to bacterial U mobilization, was investigated as a potential tool to unravel the mechanism of U mobilization and to refine the use of U isotopes as a fingerprint for subsurface processes affecting the stability of non-crystalline U(IV).

## 2. Materials and methods

### 2.1. Non-crystalline U(IV) preparation

The preparation of U(VI), i.e., the sample preparation and chemical separation, were performed in a clean laboratory environment, including extra purified acids, micro pure water (MQ-water) and precleaned PTFE beakers. The preparation of the U(VI) stock solution (IRMM-184 standard solution), the cultivation of *S. oneidensis* and the anaerobic reduction of the U(VI) to non-crystalline U(IV), the starting material of the mobilization experiments, were performed as previously described by Roebbert et al. (2021) as a modified version of that described by Stylo et al. (2013, 2015). Detailed descriptions are provided in the Supplementary Information (SI).

The biomass-associated non-crystalline U is not well described so far. U(IV) appears to be coordinated to a range of phosphate species. These species include monomers and polymerized networks associated with phosphate functional groups in the microbial biomass and species which are formed through the precipitation inorganic phosphate polymers (Alessi et al., 2014).

### 2.2. Mobilization experiment with *At. ferrooxidans*

In order to investigate the aerobic oxidation of non-crystalline U(IV) by *At. ferrooxidans* (DiSpirito and Tuovinen, 1982) two types of experiments were conducted: (I) without pre-treatment and (II) prewashed with bicarbonate to desorb any remaining U(VI). These experiments were repeated with inhibited *At. ferrooxidans* cultures, conducted by the addition of sodium formate. The inhibited cultures were used to distinguish between oxidation effects by the bacterial biomass itself and enzymatically driven oxidizing processes by the active bacteria. Additionally, abiotic control experiments were performed for all experiments, i.e., the same experimental procedure, but without any bacteria. All experiments were executed as duplicates.

**Figure 1:**
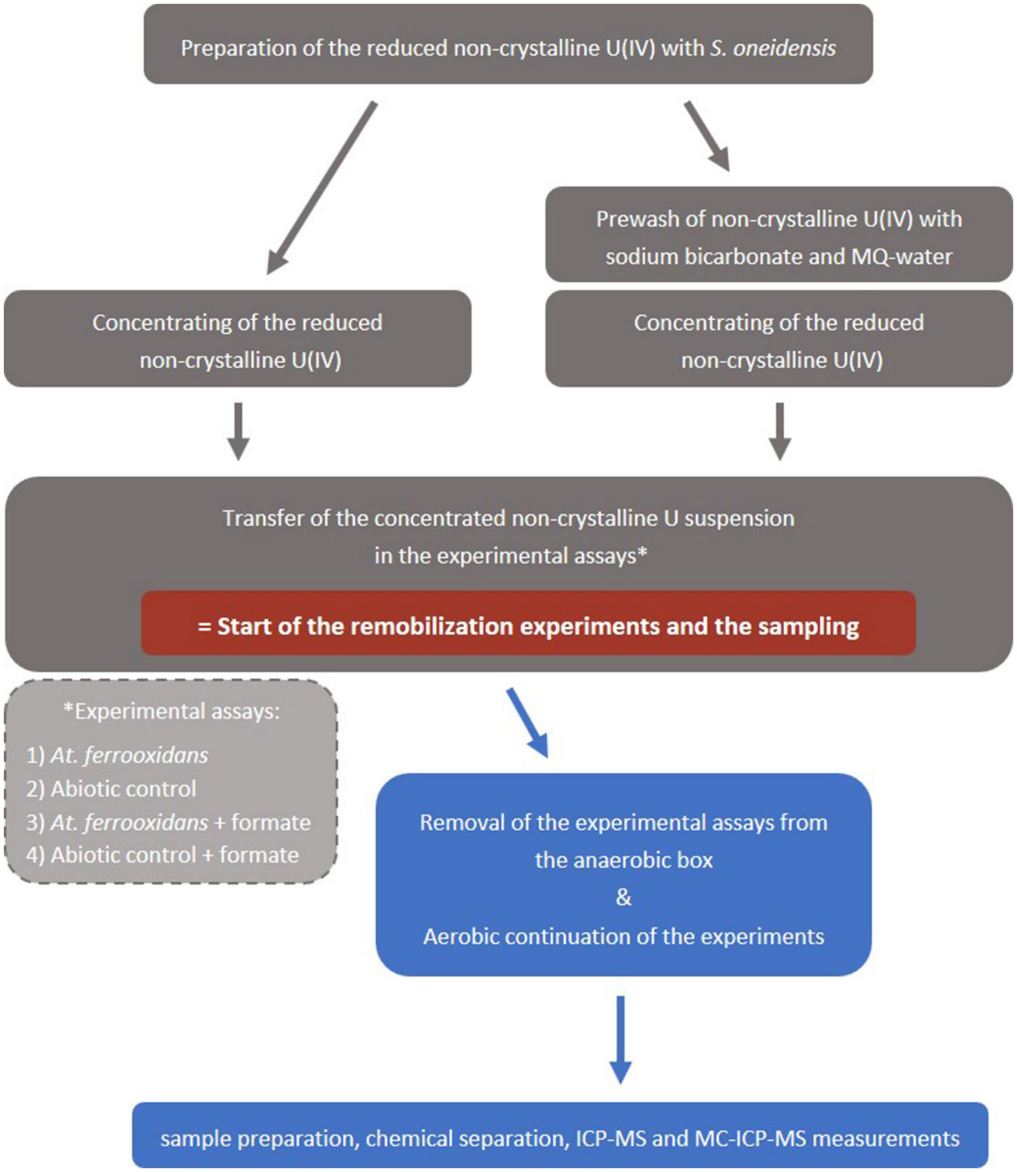
Schematic workflow of the mobilization experiments. Grey boxes 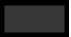 indicate anaerobic conditions and blue boxes 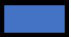 indicate aerobic conditions.

Cultivation of *At. ferrooxidans* (type strain ATCC 23270, DSM 14882) is described in the SI. The culture flasks with 50 mL *At. ferrooxidans* culture were then transferred inside an anaerobic box, where the mobilization experiments were subsequently prepared. All preparation steps were executed anaerobically to prevent premature oxidation of the non-crystalline U(IV) by oxygen. The non-crystalline U(IV) suspension (∼400 μM U) was centrifuged for 25 min at 7441 g and 21°C and the liquid was decanted. The precipitates, i.e., non-crystalline U(IV) and associated organic matter, were taken up with 3–4 mL of the *At. ferrooxidans* suspension of one culture flask and filled into the respective culture flask. This marks the start of the oxidation experiment (Figure 1) with a non-crystalline U(IV) concentration of *ca.* 240 μM (57 ppm). In case of the prewashed assays the precipitates were washed with 30 mL of 50 mM NaHCO_3_ for 1 h, before the start of the experiment. Afterwards, the bicarbonate suspension was centrifuged, the liquid phase was decanted and subsequently the precipitate was washed twice with 30 mL MQ-water before the addition of the *At. ferrooxidans* suspension (Figure 1).

After about 45 min the culture flasks with the experiment suspension were removed from the anaerobic box. The experiment continued aerobically and the suspensions were shaken up regularly, at least daily, to ensure a good mixing and a sufficient CO_2_- supply for the *At. ferrooxidans* culture.

All experiments were repeated with the addition of 0.5 mM sodium formate [Na(HCOO)] to the *At. ferrooxidans* culture before the start of the experiments. Formic acid is described as an efficient inhibitor of microbial activity for acidophile bacteria such as *At. ferrooxidans* (Zhang et al., 2020). The inhibition by formate was successfully tested, which is described in SI. Besides, formate did not react or mobilize non-crystalline U(IV) as the abiotic control experiments showed (see SI for details and Supplementary Table S6).

Sample aliquots (0.5 mL) of all experiments were withdrawn at distinct time intervals (Supplementary Tables S6, S7) and filtered through 0.2 μm nylon membranes to collect the oxidized and dissolved U(VI) fraction, whereas the solid U(IV) remained in the filter. An initial sample without filtration was taken to determine the total U concentration. The pH value (Supplementary Table S1, SI) of the bacterial culture was measured before the start of the experiment and after the start to ensure that the experiment was conducted under ideal living conditions for the bacteria. The viability of the bacteria after the addition of non-crystalline U(IV) was proven by cultivation trials (see SI for details).

### 2.3. Oxidation of non-crystalline U(IV) by *Thiobacillus denitrificans*

Cultivation of *T. denitrificans* (type strain ATCC 29685, DSM 807) is described in SI. In order to investigate the anaerobic oxidation of non-crystalline U(IV) by *T. denitrificans,* which is considered to be nitrate-dependent coupled with thiosulfate (and potentially H_2_) oxidation (Beller, 2005), three types of experiments with varying KNO_3_ and Na_2_S_2_O_3_ concentrations were performed. The experiments were modified versions after Beller (2005) in correspondence to the laboratory equipment. Abiotic control experiments for each type of experiment were performed. All experiments were executed as duplicates and in an anaerobic N_2_-environment. The prepared *T. denitrificans* suspension was split in 40 mL portions in anaerobic centrifugation tubes. After centrifugation for 20 min at 11600 g and 21°C, the cells were washed once with an anaerobic suspension buffer. The composition of the suspension buffer differed marginally from that of the growth medium. The KH_2_PO_4_ concentration was reduced to 1.5 mM to preclude the formation of soluble U(IV)-phosphate complexes (Beller, 2005) and the KNO_3_ and Na_2_S_2_O_3_ concentrations varied: 1 mM KNO_3_ (no Na_2_S_2_O_3_); 3 mM KNO_3_ (no Na_2_S_2_O_3_); 3 mM KNO_3_ + 1.9 mM Na_2_S_2_O_3_. After an additional centrifugation step, the bacterial cells were taken up with 25 mL anaerobic suspension buffer, forming ‘*T. denitrificans* suspension (2)’, and transferred into 120 mL serum bottles. In order to start the oxidation experiments the non-crystalline U(IV) and associated organic matter was taken up with 3–4 mL of the *T. denitrificans* suspension (2) of one serum bottle each and added back into the respective one. After taking the unfiltered initial sample and the start sample, the bottles were sealed with butyl rubber stoppers and 10% H_2_ and 10% CO_2_ were added to the N_2_ in the headspace (after 40 min, due to technical limitations of the anaerobic box) to simulate the atmosphere of the oxidation experiments performed by Beller (2005).

The sampling and pH (Supplementary Table S3, SI) measurement was performed as described for the *At. ferrooxidans* experiments. Additionally, the H_2_ concentration (Supplementary Table S4, SI) in the headspace was measured (via gas chromatography) at distinct time intervals, as the H_2_ consumption should serve as a proxy for U(IV) oxidation by *T. denitrificans* (Beller, 2005). The viability of the bacteria after the addition of non-crystalline U(IV) was proven by cultivation trials (see SI for details).

### 2.4. Uranium concentration and isotope analysis

The concentration measurements were carried out with a Thermo Scientific Element XR HR-ICP-MS (inductively coupled plasma mass spectrometry) equipped with an ESI SC2-DX autosampler. Iridium (Ir, 5 ng/g) was added as an internal standard. The U isotope composition was measured applying an U double spike approach (with IRMM-3636) combined with standard-sample bracketing (two samples in between standard measurements) using a Thermo-Scientific Neptune MC-ICP-MS (multi collector ICP-MS), equipped with an ESI SC2-DX autosampler, Cetac Aridus II and 100 μL min^−1^ PFA nebulizer. All isotope data are reported relative to the composition of the IRMM-184 standard.

Prior to isotope analyses, U was purified using chromatographic extraction with Eichrom UTEVA. Details about U isotope analyses are described in Weyer et al. (2008), Noordmann et al. (2015) and Roebbert et al. (2021) and are also provided in the SI. The analytical uncertainty is smaller than ±0.1 ‰.

### 2.5. Data presentation and correction

The mobilization of the non-crystalline U(IV) is displayed as the relative U concentration (c/c_0_) against time (t). The relative U concentration (c/c_0_) is defined as the ratio of the measured mobilized concentration divided by the total U concentration of the experiment. The data of the biotic mobilization experiments with *At. ferrooxidans* are control-sample-corrected. Therefore, the relative U concentrations of the abiotic control experiments of each type of experiment were pooled, i.e. arithmetically averaged for each sample time. These mean c/c_0_ derived from the abiotic controls were then subtracted from the c/c_0_ of the corresponding experiments with bacteria (active or inhibited) at the same sample time (respective data Supplementary Table S6, SI).

The isotope fractionation during the mobilization of non-crystalline U(IV) is presented using the δ notation (in ‰) against time (t). The δ value is calculated as follows:

(1) $$\delta^{238}U=\frac{{\left(\frac{U\;238}{U\;235}\right)}_{sample}-{\left(\frac{U\;238}{U\;235}\right)}_{standard}}{{\left(\frac{U\;238}{U\;235}\right)}_{standard}}*1000$$

The U isotope fractionation is displayed as the absolute δ^238^U, which means the difference between the measured δ^238^U and the initial δ^238^U before the start of the experiment (respective data Supplementary Table S5, SI).

## 3. Results and discussion

### 3.1. Biotic uranium mobilization and isotope fractionation by *Acidithiobacillus ferrooxidans*

**Figure 2:**
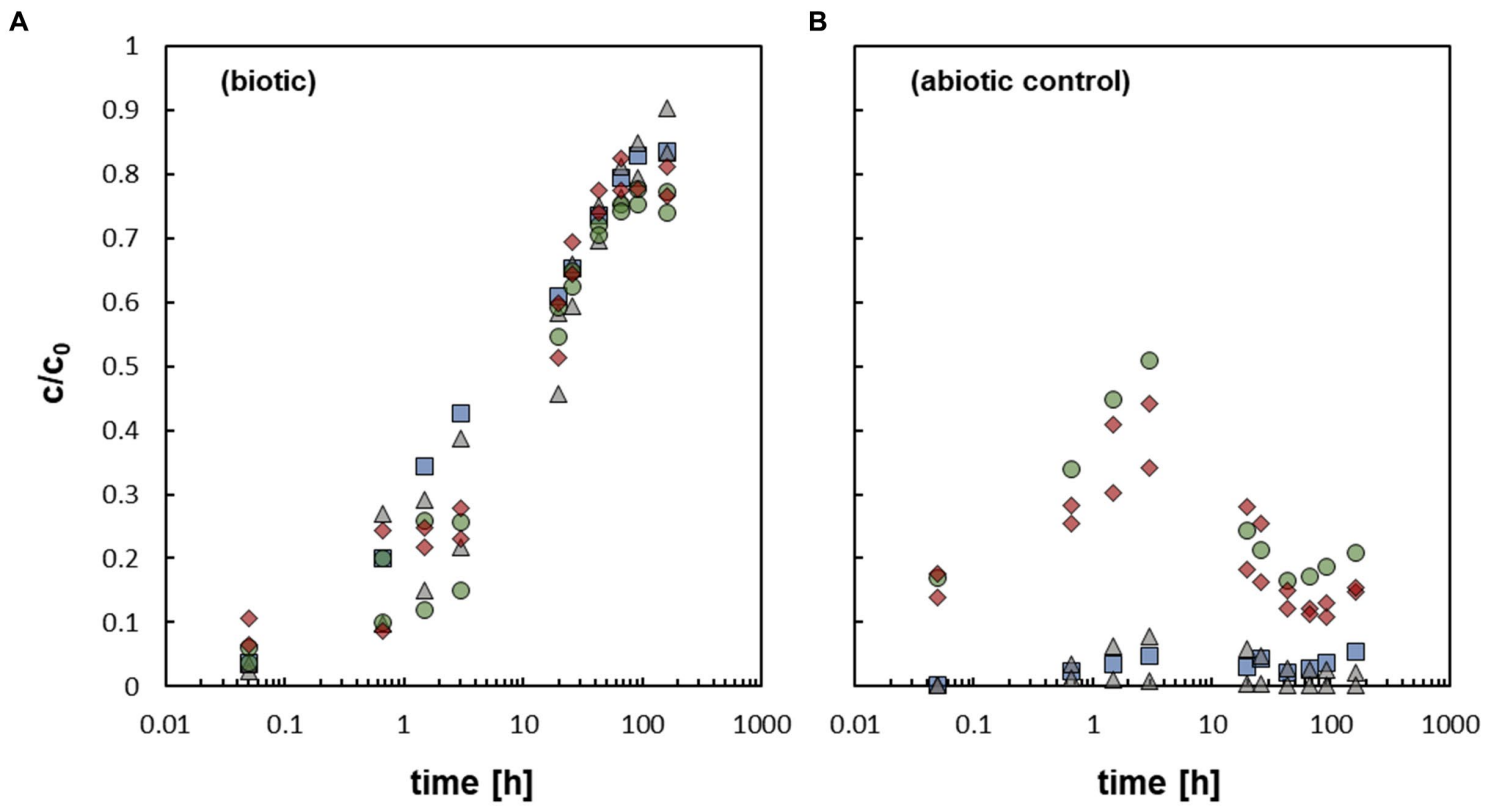
**(A)** Relative U concentration (c/c_0_) of mobilized non-crystalline U(IV) by *At. ferrooxidans* against time; control-sample-corrected (respective data Supplementary Table S6, SI). **(B)** Abiotically mobilized non-crystalline U(IV) displayed as relative U concentration (c/c_0_) against time (respective data Supplementary Table S7, SI). The different experimental setups are distinguished as follows: 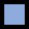 active/ untreated; 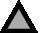 inactivated/ with formate; 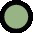 prewashed; 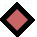 prewashed + inactivated/ formate.

The addition of the bacterium *At. ferrooxidans* caused a fast mobilization of non-crystalline U(IV) (Figure 2A). The data represent pure biotic mobilization which was calculated by subtracting the abiotic mobilization (Figure 2B) from the total mobilization (not shown) in order to eliminate abiotic mobilization effects like oxidation by dissolved oxygen. All biotic mobilization experiments showed a similar overall trend, whether with or without bicarbonate washing and/ or formate as an inhibitor of microbial activity. However, statistical dispersion of the mobilization was observed in the first 3 h, but in fact independently of the experimental setup. The data imply that the effect of the biomass of the bacterial cells is more important than metabolic processes of the active cells.

In all samples a fast increase in c/c_0_ in the first 3 h could be observed, which subsequently slowed down until a maximum c/c_0_ of 75–90% was reached after 91.75 h to 164 h. In additional cultivation experiments (see SI for details), it could clearly be shown that formate is an effective inhibitor of microbial activity of *At. ferrooxidans* (Zhang et al., 2020), although it did not inhibit the mobilization of non-crystalline U(IV). Likewise, it could be demonstrated that the bacterial cells were not harmed by the addition of 400 μM non-crystalline U(IV) as they showed similar activity and growth when subsequently cultivated on fresh medium. In order to explain the similar mobilization of non-crystalline U(IV) observed in the experiments with active and inhibited cells various mobilization options are discussed.

### 3.2. Transportation and energy-yielding processes

First, transportation and energy-yielding processes may play a role in the mobilization of cations, but they are unlikely to be the reason for the mobilization of non-crystalline U(IV) by *At. ferrooxidans*. Active transport of solutes inside cells can be realized by simple transport, group translocation and ABC transport system, whereby transported substances can be modified chemically in terms of group translocation (Quatrini and Johnson, 2016). However, it is known that iron-containing solid substrates, e.g. FeS_2_ cannot enter the *At. ferrooxidans* cells. Instead the Fe(II) is oxidized on the outer cell membrane outside the cell (Valdés et al., 2008). This probably is transferrable for the sparingly soluble non-crystalline U(IV), and thus oxidation of non-crystalline U(IV) via transportation processes is unlikely, but this does not preclude a potential uptake of soluble U(IV) inside the cell.

It is assumed that *At. ferrooxidans* is able to oxidize U(IV) either directly or indirectly by an oxidation–reduction reaction with Fe^3+^ (DiSpirito and Tuovinen, 1981). Direct oxidation can be ruled out as the translocation of electrons depends on an energized membrane. Formic acid, which was used to inhibit the microbial activity, permeates across microbial cell membranes at low pH into the cytoplasm where the formic acid dissociates and thereby decreases the internal pH. Thereby, intracellular metabolic reactions and energizing of the membrane are inhibited (Lambert and Stratford, 1999; Ballerstedt et al., 2017; Luise et al., 2020). Thus, redox reactions are unlikely to happen as there is no energetic imbalance. Indirect oxidation by an oxidation–reduction reaction relies on Fe^3+^ as the oxidizing agent for U(IV) followed by the oxidation of the resulting Fe^2+^ by *At. ferrooxidans*, meaning ferric-ferrous iron recycling (Harrison et al., 1966; Kalinowski et al., 2004). This indirect process can be ruled out as well. Due to the different amounts of U (12 μmol) and Fe (0.1 μmol for doping of the growth medium) in this study and a stoichiometric ratio of 1:2 (U^4+^ + 2Fe^3+^ ➔ U^6+^ + 2Fe^2+^) the Fe would need to run the ferric-ferrous iron cycle at least 180 times at ideal conditions in order to oxidize 75% of the U(IV). Even so, the inactive cells are not capable of running the ferric-ferrous iron cycle because of the inhibition of intracellular metabolic reactions by formate. Even if assuming that all Fe is present as Fe^3+^ it can oxidize only about 0.4% of the U(IV) at a maximum and thus can be neglected.

In order to increase the reception of free energy by oxidizing inorganic compounds most cellular reactions depend on enzyme-catalysis to increase the rate of reaction, and the same applies to *At. ferrooxidans* (Quatrini and Johnson, 2016). However, the bacterial cells depend on an energized membrane, which is lacking in inhibited bacterial cells, in order to carry out enzyme-catalyzed reactions. Thus, these are ruled out as well as reasons for the mobilization of non-crystalline U(IV) observed in this study.

### 3.3. U mobilization options: biosorption, complexation, and oxidation processes by biomass or released chemical compounds

More promising explanations for the mobilization of non-crystalline U(IV) might be biosorption and/ or complexation and oxidation processes by the biomass itself. Biosorption is defined as the ability of microbial cells to sequester heavy metals and radionuclides selectively from aquatic solutions to the cell surface via non-metabolically mediated pathways (Tsezos, 2014). Therefore, both, living and dead cell biomass is capable of biosorption. The U biosorption is controlled by the chemistry and pH of the solution, physiological state of the cells as well as the presence of soluble polymers (Merroun and Selenska-Pobell, 2008). The cell wall of *At. ferrooxidans* is made up of a thin peptidoglycan layer protected by the lipid/protein bilayer of their outer membrane. Lipopolysaccharides (LPS) are anchored to the outer membrane and are probably the preferred centers of cation binding as they contain phosphate and frequently also carboxylate groups (Merroun and Selenska-Pobell, 2008). Biosorption of U(IV) by *At. ferrooxidans* might be a possible explanation for the mobilization of non-crystalline U(IV) if the latter forms soluble compounds, as biosorption of several other metals, e.g. Cu, Zn, As, and Mo, has already been observed (Ruiz-Manríquez et al., 1998; Celaya et al., 2000; Yan et al., 2010; Kasra-Kermanshahi et al., 2021). However, although being metabolism-independent, biosorption is limited to the number of charged groups within the surface layers of the cell (Merroun and Selenska-Pobell, 2008). Moreover, the estimated ion surface area exceeds the cell surface area by several magnitudes. If the almost rod shaped *At. ferrooxidans* cells are mathematically approximated with a cylinder of 1 μm length and 0.5 μm diameter, a total surface area of 9.8 **·** 10^8^ μm^2^ per experimental assay is obtained, with 10^7^ cells per ml and 50 mL culture. On the other hand, the estimated minimum required surface area for all uranium ions is 1.22 **·** 10^17^ μm^2^ [area of a disk, 0.89 pm minimum ion radius (Shannon, 1976), U concentration of 40 μg/g (Supplementary Table S6) per 50 mL experimental assay at the minimum after 164 h]. Thus, biosorption onto the cell as the main U mobilization mechanism is very unlikely.

In addition, the mobilization of non-crystalline U(IV) observed in this study has similarities to the complexation and associated mobilization of non-crystalline U(IV) by organic ligands, such as EDTA, citrate and bicarbonate described by Roebbert et al. (2021). During the complexation of non-crystalline U(IV) soluble U compounds were formed. Bicarbonate was used in some experiments of this study as well, in order to detach remaining U(VI) after the reduction process. The increased abiotic mobilization of non-crystalline U(IV) in the experiments with bicarbonate prewashing could be explained by differences in the pH of the experiments. In the abiotic experiments with prewashing the pH remained around 2.5 after the start of the experiments, whereas without prewashing the pH increased to 3.29–3.73 (Supplementary Table S1, SI). Torrero et al. (1997) showed a decreasing dissolution of UO_2_ with increasing pH, which is probably transferable to non-crystalline U(IV). Based on the repeated washout with MQ water, it can be assumed that no more bicarbonate was present in the system. A potential effect of the bicarbonate prewashing on the biotic U(IV) mobilization, observed in this study, was eliminated by subtracting the mobilization observed for abiotic reference experiments (averaged for each time point) from that of the respective biotic experiments. Additionally, the experiments with and without prewashing had similar biotic mobilization rates.

Simple oxidation of the non-crystalline UIV) by oxygen can be ruled out as well as the experimental assays already showed fast U mobilization in anaerobic conditions during the first 45 min. Any oxygen effects after the removal from the anaerobic box would have also influenced the abiotic controls, and therefore would have been control-sample-corrected in the *At. ferrooxidans* dataset.

Complexation mechanisms of the cell components and cell surface itself are complemented by complexation processes with extracellular polymeric substances (EPS). Decho (2011) defines EPS as molecules having a range of sizes, compositions and chemical properties that are produced and secreted (i.e. they are extracellular) by bacteria and other microorganisms, and contribute to the cell adaptability, resiliency, and functional roles in environments. Regarding *At. ferrooxidans,* it is known that the cells are able to adapt the chemical composition of their exopolymers to the substrate. Its EPS mainly consist of sugar, lipids, nitrogen and phosphorous with varying proportions (Gehrke et al., 2001). Merroun et al. (2003) demonstrated that U is associated to the acidic EPS of *At. ferrooxidans.* U complexes are suggested to be structurally similar to U-fructose-phosphate-complexes obtained by Koban et al. (2004) indicating that organic phosphate groups of EPS might play a leading role in the complexation processes and thus possibly forming soluble complexes.

It cannot be determined from the results whether or not the non-crystalline U(IV) is oxidized during the mobilization process, although oxidation might be energetically preferable. The mobilization might be driven by either a specific mechanism or a mixture of different mobilization processes. However, the results clearly show that inactive *At. ferrooxidans* cells are capable of mobilizing non-crystalline U(IV). The biomass of the bacterial cells is probably the driving factor for the mobilization, which stands in contrast to the hypothesis of direct oxidization of U(IV) in connection with carbon dioxide fixation by DiSpirito and Tuovinen (1981, 1982). The complexation hypothesis of this study is supported by the results of the complexation of non-crystalline U(IV) by organic ligands, especially EDTA and bicarbonate, described by Roebbert et al. (2021), which showed similar mobilization rates to the experiments with *At. ferrooxidans* with organic ligands. The slightly lower maximum c/c_0_ can simply be explained by a difference of mobilization by different ligands and a higher initial non-crystalline U(IV) concentration used by Roebbert et al. (2021) (400 μM) compared to that in this study (240 μM). However, the mobilization by EDTA, citrate and bicarbonate showed U isotope fractionation in contrast to the mobilization observed in our study. There are several explanations for the differences in the isotope fractionation, which are discussed hereafter.

### 3.4. Isotope fractionation during U mobilization with *At. ferrooxidans*

**Figure 3:**
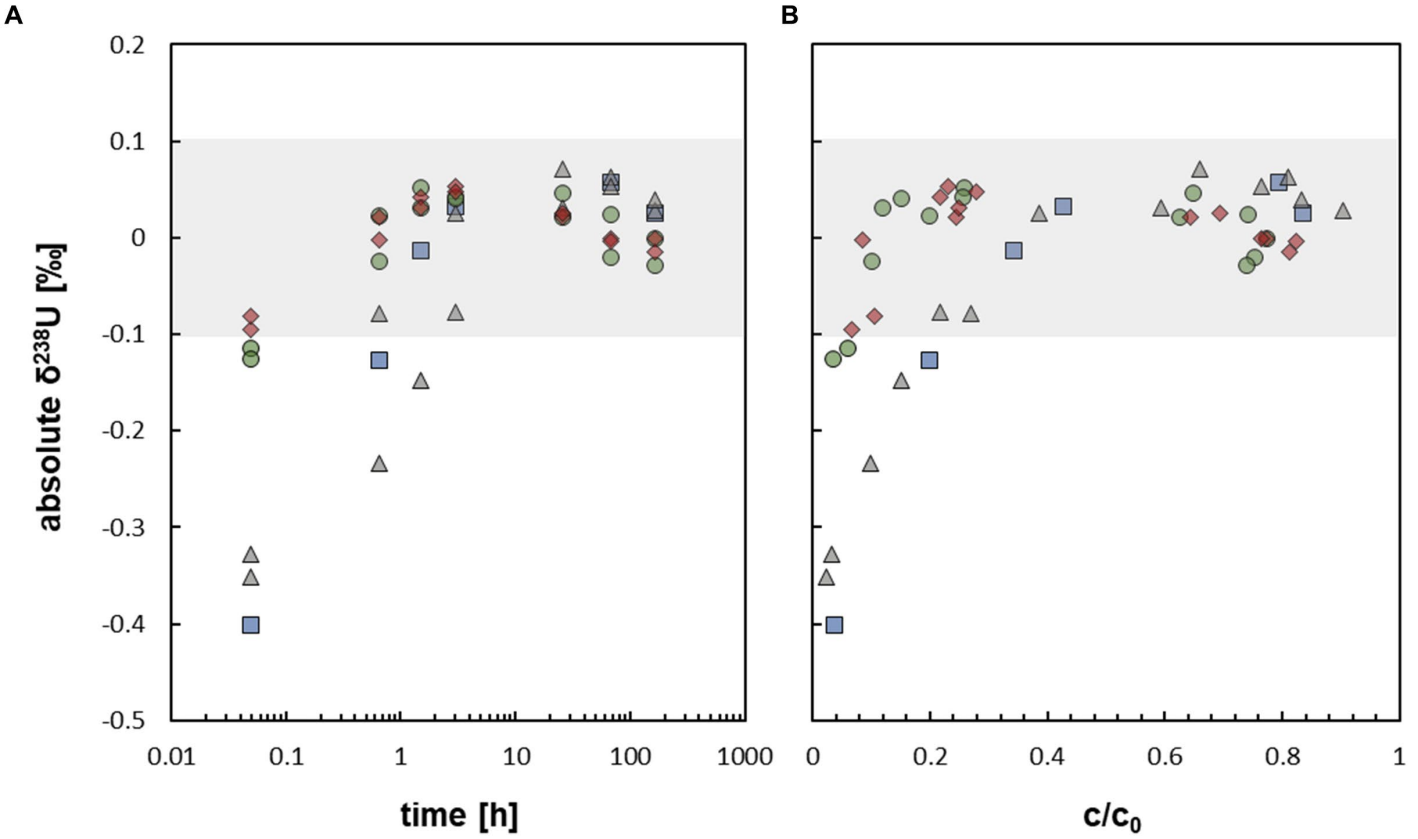
**(A)** Uranium isotope fractionation displayed as absolute δ^238^U [‰] against time during the mobilization of non-crystalline U(IV) by *At. ferrooxidans* (respective data Supplementary Table S5, SI). **(B)** Uranium isotope fractionation displayed as absolute δ^238^U [‰] against relative U concentration c/c_0_ during the mobilization of non-crystalline U(IV) by *At. ferrooxidans* (respective data Supplementary Tables S5, S6, SI). The grey field 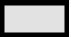 displays the max. Analytical uncertainty of ±0.1 ‰ (for all samples). The different experimental setups are distinguished as follows: 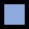 active/ untreated; 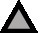 inactivated/ with formate; 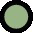 prewashed; 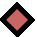 prewashed + inactivated/ formate.

During the mobilization experiments with *At. ferrooxidans*, conducted in this study, the isotopic composition of U approximated δ^238^U ∼ 0 ‰ after about 1.5 h, when about 20 to 30% of U was mobilized (Figures 2A, 3A,B). Thus, no net isotope fractionation occurred during U mobilization, within the analytical uncertainty of ±0.1 ‰. The experiments without bicarbonate prewashing showed early isotope fractionation towards low δ^238^U (−0.40 ‰ to −0.32 ‰ after 0.05 h increasing to ≤ −0.15 ‰ after 1.5 h) which, however, subsequently increased towards δ^238^U approximating ∼ 0 ‰. The experiments with bicarbonate prewashing showed early isotope fractionation of δ^238^U = −0.12 ‰ resp. -0.13 ‰, barely outside the analytical error (Figure 3A). The initial light isotopic composition may be explained by preferential mobilization of remaining U(VI) from the reduction process, as observed by Roebbert et al. (2021) for, e.g. the organic ligand citrate. Although, reduction of U(VI) by *S. oneidensis* MR-1 is efficient to produce non-crystalline U(IV), which was used as the experimental starting material, some U(VI) may have remained (Stylo et al., 2015). During the reduction process the heavier ^238^U is primarily reduced, resulting in a very light δ^238^U of the remaining U(VI) (Stylo et al., 2015). This effect of initially light δ^238^U was impeded with a bicarbonate prewash step (Roebbert et al., 2021) which appears to have effectively removed almost all remaining U(VI).

The absence of U isotope fractionation during the mobilization of non-crystalline U(IV) by *At. ferrooxidans*, although complexation of non-crystalline U(IV) by organic ligands and oxidation or mobilization of other transition metals, i.e. Fe and Cu, by *At. ferrooxidans,* resulted in significant isotope fractionation (Balci et al., 2006; Kimball et al., 2009), may have several reasons which are outlined below.

For U, large isotope effects are considered to be associated with redox-reactions (Weyer et al., 2008). In previous experiments it was observed that light U isotopes were preferentially oxidized from dissolved U(IV) in acidic media, resulting in U(VI) being approx. 1.1 ± 0.2 ‰ lighter than the remaining U(IV) (Wang et al., 2015a, b). In contrast, only limited U isotope fractionation (δ^238^U ∼ 0.1–0.3 ‰ in dissolved U(VI)) was observed by Wang et al. (2015a, b) during oxidation of uraninite by dissolved oxygen in 20 mM NaHCO_3_ solution at pH = 9.4. The authors explained the absence of significant U isotope fractionation by a so-called “layer-effect” during which quantitative dissolution of each layer of uraninite occurs, impeding net isotope fractionation.

However, complexation of U, which, as discussed beforehand, probably is an important mechanism during the mobilization process in this study, does not exclusively require electron transition, i.e. redox processes. Complexation can also be caused by electrostatic interaction or due to sharing of electrons, i.e. without changing the oxidation number of U. Nevertheless, significant U isotope fractionation was observed during the complexation of non-crystalline U(IV) by organic ligands at anoxic conditions (δ^238^U ∼ 0.2–0.7 ‰) (Roebbert et al., 2021), even though no U redox change was observed.

Likely, U mobilization and resulting isotope fractionation during our experiments was driven by a sequence of consecutive processes which occurred at different time scales. At the beginning of the experiments, remaining isotopically light U(VI) was mobilized, resulting in the initially observed light U isotope compositions within the first 1–3 h. This signature was subsequently superimposed by that of mobilized non-crystalline U(IV) which may have occurred in a chain of reaction steps, e.g. involving U adsorption onto cell biomass, followed by U complexation and/or oxidation by compounds of the biomass of *At. ferrooxidans*. Such processes might cause isotope fractionation in opposite directions which may superimpose or even neutralize each other within the term of the experiment. Likewise, the initial step of the reaction chain, i.e. U adsorption onto the biomass, was slower than the following steps and generated no detectable isotope fractionation. In this case, any potentially generated isotope fractionation of the following steps, including U complexation and/or oxidation by the biomass, would essentially go to completion and thus, not significantly contribute to the observed net isotope fractionation. Furthermore, in closed systems the U isotope signature of the mobilized U must approach 0 ‰ relative to that of the starting value with increasing U mobilization. Thus, small isotope effects would not be resolvable anymore, when a significant part of U was mobilized already (e.g. 50–80%, after 3 h).

### 3.5. Uranium mobilization and isotope fractionation by *Thiobacillus denitrificans*

No mobilization of non-crystalline U(IV) by *T. denitrificans* could be observed. Furthermore, by cultivating *T. denitrificans* on fresh medium inoculated from the oxidation experiments, it could be demonstrated that the bacterial cells remained alive during the experiment, although the number of bacterial cells observable in the experimental assay seemed to have decreased with time. The pH of the experimental suspension was constantly in the pH range of 6.9–8.2 (Supplementary Table S3, SI), optimal for the growth of *T. denitrificans* (Kelly and Wood, 2000).

Several modifications of the experiment described by Beller (2005) had to be done in order to adapt the experimental procedure to the available equipment of the laboratory. Slight differences in the cultivation procedure, recommended by the DSMZ, were a late addition of 10% (v/v) H_2_, addition of 10% (v/v) CO_2_ to the atmosphere of the set-up and a larger volume of suspension buffer. These modifications might have influenced the activity of the bacterial cells and their ability to adapt to U as a potentially new energy source. Furthermore, Beller (2005) used synthetic U(IV), which was derived from uranyl acetate, and biogenic uraninite. *T. denitrificans* might simply not be able to mobilize the non-crystalline U(IV) used in our study, although this is unlikely, because the non-crystalline U(IV) is considered to be more labile than uraninite (Bernier-Latmani et al., 2010). The mobilization by *T. denitrificans* also might be compensated by a withdrawal of mobilized U. The mobilization of U(IV) is presumably slower than the mobilization by *At. ferrooxidans* (Beller, 2005). The apparent lack of mobilization may also be explained by different strains of *T. denitrificans* (Beller, 2005). The ability of a bacterium to perform a certain mechanism can change, as the bacteria can adapt to different environmental conditions over several generations or a loss of their genetic ability due to selection pressure (Suzuki et al., 1990; Tang et al., 2006; Sunwoo et al., 2013; Zhang et al., 2013).

## 4. Conclusions and implications for long-term feasibility of *in-situ* U bioremediation measures and the use of U isotope proxy

The capability of *At. ferrooxidans* to mobilize non-crystalline U(IV) could be demonstrated, independently from the presence of active bacterial cells. Experimental assays with active *At. ferrooxidans* cultures showed similar U mobilization rates like the assays with inhibited bacterial cells. Complexation and/ or oxidation processes by biomass are probably the driving factors for the mobilization. This finding has important implications for the understanding of U remobilization and for the long-term feasibility of *in-situ* remediation sites of U contamination, as other types of bacteria may show similar effects of mobilization of non-crystalline U, simply by their biomass. As the acidic conditions of these experiments are far from natural conditions in the groundwater, the findings of this study are only directly applicable for acid mine drainage and bioleaching sites. Nevertheless, the high efficacy and insensitivity of the U mobilization capacity of *At. ferrooxidans* on the portion of living cells raises doubts on the long-term sustainability of *in-situ* bioremediation measures from acidic environments.

During the mobilization experiments with *At. ferrooxidans* no net isotope fractionation was observed, except for early isotope fractionation in the experiments without bicarbonate prewashing, which can be explained by preferential mobilization of remaining U(VI) from the preparation process. The U mobilization and resulting isotope fractionation was likely driven by a sequence of consecutive processes which occurred at different time scales and probably superimposed each other to some extent. According to the findings of this study, U isotope signatures appear to be an unsuitable tool to trace oxidative U mobilization by bacteria in the subsurface environment, even though comparative data of other bacterial systems are lacking. However, this may preserve U isotope signatures, which could provide information about, e.g. the source of the uranium contamination. Uranium isotopes signatures found in nature are more likely to be generated by other processes such as U reduction, adsorption, or mobilization with ligands.

## Data availability statement

The original contributions presented in the study are included in the article/Supplementary material, further inquiries can be directed to the corresponding authors.

## Author contributions

CR, YR, AS, and SW contributed to conception and design of the study. CR and YR performed the sample analysis. CR organized the database, performed the statistical analysis and wrote the first draft of the manuscript. YR wrote sections of the manuscript. All authors contributed to the article and approved the submitted version.

## Funding

This work was funded by the DFG/SNSF grants (WE 2850-16/1 and 200021E-164209: Fate of tetravalent uranium under reducing conditions).

## Conflict of interest

The authors declare that the research was conducted in the absence of any commercial or financial relationships that could be construed as a potential conflict of interest.

## Publisher’s note

All claims expressed in this article are solely those of the authors and do not necessarily represent those of their affiliated organizations, or those of the publisher, the editors and the reviewers. Any product that may be evaluated in this article, or claim that may be made by its manufacturer, is not guaranteed or endorsed by the publisher.

## Supplementary material

The Supplementary material for this article can be found online at: https://www.frontiersin.org/articles/10.3389/fmicb.2023.1190962/full#supplementary-material
