# Supplementary material for: U mobilization and associated U isotope fractionation by sulfur-oxidizing bacteria
Source: Front Microbiol. 2023 Jul 18;14:1190962. doi: 10.3389/fmicb.2023.1190962 (PMC10390777; doi:10.3389/fmicb.2023.1190962)
Supplement: Supplementary file 1 [file Data_Sheet_1.pdf]

Supporting Information

1.1 Bacteria cultivation

S. oneidensis, At. ferrooxidans and *T. denitrificans* were cultivated at sterile conditions. Cell numbers were determined by counting of cells in a Thoma chamber under a light microscope with phase contrast and 400x magnification.

1.1.1 *Shewanella oneidensis* MR-1

The protocol for cultivation of *Shewanella (S.) oneidensis* MR-1 was a modified version after Stylo et al. (2013). The bacteria were grown aerobically in 50 ml Luria-Bertani medium (LB-medium) in 250 ml culture flasks with chicanes in order to enhance the gas-liquid mass transfer while mixing on the shaker. The LB-medium was a complex growth medium and, in this case, consisted of 25 g LB-powder per 1 L micro pure water (MQ-water). The microbial strain was stored at -80 °C. After thawing the bacterial culture, 150 µl of the culture were added to 50 ml medium. The cultivation lasted for 18 hours at 30 °C on a shaker with 120 rpm, resulting in an optical density of the culture of OD_{600} ~ 2. This means the bacterial culture was at the beginning of its stationary phase ensuring an almost constant number of bacteria.

1.1.2 *Acidithiobacillus ferrooxidans*

Acidithiobacillus (At.) ferrooxidans (type strain ATCC 23270, DSM 14882) was grown in 50 ml basal salt medium (Wakeman et al., 2008; German Collection of Microorganisms and Cell Cultures GmbH: *Acidithiobacillus ferrooxidans* DSM 14882). Additionally, 1 ml trace elements were added with the following composition (g/L): $ZnSO_4 \cdot 7H_2O$ (10), $CuSO_4 \cdot 5H_2O$ (1.0), $MnSO_4 \cdot 4H_2O$ (1.0), $CoSO_4 \cdot 7H_2O$ (1.0), $Cr_2(SO_4)_3 \cdot 15H_2O$ (0.5), H_3BO_3 (0.6), $Na_2MoO_4 \cdot 2H_2O$ (0.5), $NiSO_4 \cdot 6H_2O$ (1.0), $Na_2SeO_4 \cdot 10H_2O$ (1.0), $Na_2WO_4 \cdot 2H_2O$ (0.1) and $NaVO_3$ (0.1). The medium was adjusted to pH 2.5 with sulfuric acid and sterilized in an autoclave for 15 min at 121°C. As growth substrate, 10 µl of a filter sterilized 10 mM $FeSO_4$ solution was added. For cultivation experiments in presence of formate, 1 % elemental sulfur was supplied, previously sterilized by tyndallisation. For U mobilization experiments, the elemental sulfur in the growth substrate was reduced to 0.05% (0.025 g in 50 ml) in order to minimize a possible interaction with the non-crystalline U(IV). 100 ml culture flasks with 50 ml medium were incubated with 1 ml (2 %) inoculated microbial culture for 5 days at 30 °C on a shaker with 120 rpm, resulting in a slight turbidity. 10^6 to 10^7 cells per ml were counted after 5 days. The microbial strain, which originally grew on ferrous sulfate as the only substrate, was adapted to grow on sulfur by several transfers to fresh medium with subsequent cultivation.

Table S1 | pH values before and after the start of the mobilization experiments with *At. ferrooxidans*

Experiment	pH (before start)	pH (after 5 min)
**Biotic (1:1)**		
active/ untreated (1)	1.96	2.08
active/ untreated (2)	2.10	2.51
prewashed (1)	1.92	1.92
prewashed (2)	2.00	2.00
inactivated/ with formate (1)	2.02	2.17
inactivated/ with formate (2)	1.99	2.32
prewashed + inactivated/ formate (1)	1.97	1.97
prewashed + inactivated/ formate (2)	1.91	1.98
**Abiotic (control sample)**		
untreated	2.52	3.35
prewashed	2.45	2.60
with formate (1)	2.59	3.29
with formate (2)	2.48	3.73
prewashed + formate (1)	2.55	2.66
prewashed + formate(2)	2.58	2.61

1.1.3 *Thiobacillus denitrificans*

The cultivation of *Thiobacillus (T.) denitrificans* (type strain ATCC 29685, DSM 807) was performed after the instruction of the DSMZ (German Collection of Microorganisms and Cell Cultures GmbH: *Thiobacillus denitrificans* DSM 12475). For the growth medium, four solutions (A, B, C and D, Table S2) were prepared and sterilized.

Table S2 | Compositions of the cultivation solutions A, B, C, D

**Solution A**			total growth medium
KH_2PO_4	2.0	g	14.7 mM
KNO_3	2.0	g	19.8 mM
NH_4CL	1.0	g	18.7 mM
$MgSO_4$ * 7 H_2O	0.8	g	3.2 mM
Trace element solution SL-4	2.0	ml	----
Distilled water	960.0	ml	----
(adjust pH to 7.0 with NaOH)			
**Solution B**			
$Na_2S_2O_3$	5.0	g	20.1 mM
Distilled water	20.0	ml	----
**Solution C**			
$NaHCO_3$	1.0	g	11.9 mM
Distilled water	20.0	ml	----
**Solution D**			
$FeSO_4$ * 7 H_2O	2.0	mg	0.07 mM
0.1 N H_2SO_4	1.0	ml	----

Trace element solution SL-4		
Na_2-EDTA	0.50	g
$FeSO_4$ * 7 H2O	0.20	g
$ZnSO_4$ * 7 H_2O	0.10	g
$MnCl_2$ * 7H_2O	0.03	g
H_3BO_3	0.30	g
$CoCl_2$ * 6 H_2O	0.20	g
$CuCl_2$ * 2 H_2O	0.01	g
$NiCl_2$ * 6H_2O	0.02	g
Na_2MoO_4 * 2H_2O	0.03	g

(first dissolve EDTA in distilled water, then adjust pH to 7.0 with 2 M NaOH and afterwards add the other compounds)

The solutions A, B and D were weighed and transferred into bottles with rubber plug screw tops and afterwards sterilized separately in an autoclave at 121 °C for 15 min. After removing the bottles from the autoclave at a temperature of 90 °C, the solutions were immediately flushed with N_2 for 3 hours to achieve anaerobic conditions. In order to prepare solution C, the $NaHCO_3$ was also weighed and transferred into a bottle with a rubber plug screw top and rinsed with N_2 for 30 min. Afterwards, it was dissolved in anaerobic, distilled water inside an anaerobic chamber under a N_2-atmosphere and the solution was sterilized by filtration. All solutions were mixed inside an anaerobic chamber to prepare the final growth medium. For the cultivation of *T. denitrificans*, the growth medium and 2 % of the microbial culture (e.g. 200 ml growth medium and 4 ml microbial culture) were given into a serum bottle and kept at 30°C for 2 – 3 days. The number of bacterial cells was 10^8 cells ml^{-1}, i.e. the culture was in the late exponential growth phase (Beller, 2005).

Table S3 | pH values before and after the start of the mobilization experiments with *T. denitrificans*

Experiment	pH (before start)	pH (after 5 min)	pH (after 3 d)	pH (after 7 d)	pH (after 24 d)
**3 mM KNO_3 (no $Na_2S_2O_3$)**					
biotic (1)	7.00	8.08	7.00	7.08	7.02
biotic (2)	7.00	8.09	7.13	7.11	7.10
biotic + formate (1)	7.00	8.08	6.93	7.06	7.05
biotic + formate (2)	7.00	8.07	7.05	7.20	7.06
abiotic	7.00	7.96	7.12	7.14	7.22
abiotic + formate	7.00	7.94	7.14	7.24	7.20
**3 mM KNO_3 + 1.9 mM $Na_2S_2O_3$**					
biotic (1)	7.00	8.03	7.07	7.24	6.96
biotic (2)	7.00	7.99	7.15	7.19	6.95
biotic + formate (1)	7.00	8.17	7.09	7.33	6.98
biotic + formate (2)	7.00	8.14	7.13	7.41	6.97
abiotic	7.00	8.05	7.08	7.44	7.08
abiotic + formate	7.00	8.07	7.18	7.34	6.95

Table S4 | Development of the H_2-concentration (%) in the headspace during the oxidation experiments with *T. denitrificans*

Experiment	Start H_2 [%]	H_2 after 4 d [%]	H_2 after 28 d [%]
**3 mM KNO_3 (no $Na_2S_2O_3$)**			
biotic (1)	9.5	9.4	7.7
biotic (2)	1.3	11.9	9.4
biotic + formate (1)	9.9	9.7	8.7
biotic + formate (2)	9.9	9.9	8.5
abiotic	9.5	9.5	7.2
abiotic + formate	8.8	9.0	7.3
**3 mM KNO_3 + 1.9 mM $Na_2S_2O_3$**			
biotic (1)	8.7	7.9	8.2
biotic (2)	9.7	8.8	9.3
biotic + formate (1)	10.9	9.5	10.1
biotic + formate (2)	10.0	9.2	9.8
abiotic	9.9	9.8	9.7
abiotic + formate	9.8	9.7	9.9

Cultivation of *Acidithiobacillus ferrooxidans* from the U mobilization experiment

In order to prove the viability of the bacteria after the addition of non-crystalline U(IV), the bacteria were cultivated from a suspension equal to the performed mobilization experiments without sodium bicarbonate prewashing. Therefore, two culture flasks with 50 ml bacteria suspension with 0.05 % S were prepared as described above. Then, the non-crystalline U(IV) was added to one of the culture flasks as described in the cultivation instruction and the other one was left without U as the reference suspension. After 3 and 7 days, the number of cells in both suspensions was counted and a new culture flask with 50 ml fresh medium with 0.05 % S was inoculated with 1 ml of the bacteria suspension with U, respectively. Again, samples were taken after 3 days and 8 days, and the number of cells was counted each.

The initial cell concentration before the start of the experiment was 10^7 cells ml^{-1} in the bacterial suspension. 3 days after the inoculation the flask without U contained 1.6 * 10^7 cells ml^{-1} and the flask with U 0.9 * 10^7 cells ml^{-1}. After 7 days the flasks contained 1.9 * 10^8 cells ml^{-1} (without U) and 1.9 * 10^7 cells ml^{-1} (with U). The newly inoculated medium (3 days after start) contained 6.3 * 10^6 cells ml^{-1} after 3 days and (inoculated 7 days after start) 3.6 * 10^7 cells ml^{-1} after 8 days. Thus, the integrity of the bacteria despite the addition of non-crystalline U could be confirmed.

Cultivation of *Thiobacillus denitrificans* from the U mobilization experiment

In order to investigate the viability of the *T. denitrificans* cells at the end of the mobilization experiment after 3 weeks, three 50 ml serum bottles were prepared with 25 ml of the growth medium each and inoculated with 1 ml bacterial culture each in an anaerobic chamber. The bacterial culture was taken from serum bottles from the mobilization experiment with 3 mM KNO_3 and 3 mM KNO_3 + 1.9 mM $Na_2S_2O_3$ (see manuscript). The serum bottles were kept at 30°C for 3 days.
No differences between the original microbial culture and the cultures, which were inoculated from the experimental assays could be identified optically. The number of counted bacterial cells was about 10^8 cells ml^{-1} in all cultures. Thus, the viability of the bacteria was confirmed.

Cultivation tests of *Acidithiobacillus ferrooxidans* with 0.5 mM sodium formate

In order to distinguish between mobilization effects by only the biomass of the bacteria from those by enzymatic processes of active bacteria, 0.5 mM sodium formate (Na(HCOO)) was added to several culture flasks of the mobilization experiments to inhibit the bacteria (Zhang et al., 2020). The inhibitory effect of formic acid was tested for *At. ferrooxidans* adapted to sulfur. *At. ferrooxidans* was cultivated as described above (with 1 % S and 25 ml growth medium and 1 ml bacterial culture in serum bottles), either with 0.5 mM formate or without formate. Additionally, an experiment with *At. ferrooxidans* previously adapted to $FeSO_4$ in 100 ml culture flasks with 50 ml basal salt medium, 500 µl of a 1 M $FeSO_4$ solution and 1 ml culture was performed with and without formate. After 5 days, samples were taken and the bacteria were examined and compared via microscopy.
In the cultivation test with sulfur the suspension without formate was milky and most of the sulfur descended. In contrast, the solution with formate was clear colorless and less sulfur descended. In the cultivation test with $FeSO_4$ the color of the suspension without formate changed to orange-brown, which is typical for bacterial oxidation of Fe^{2+} to Fe^{3+}. In contrast, the solution with formate remained milky, but colorless like both assays appeared at the beginning. The suspensions without formate had more than 10^6 cells ml^{-1}, and in the solutions with formate no cells could be observed.

2.5 Sample preparation and chemical separation

The samples of the mobilization experiments were analyzed by ICP-MS and MC-ICP-MS to determine the U concentration and U isotope composition. Regarding the concentration measurement the samples were diluted ca. 1:1000 with 3 % HNO_3 with 5 ppb iridium (Ir) which was used as an internal standard.

In order to analyze the U isotope fractionation several preparation steps were necessary. At first, the samples were weighed in screw-top Savillex beakers (15 ml). Then, 4 ml concentrated aqua regia (3 ml 11 M HCl + 1 ml 14 M HNO_3) were added and the closed beakers were kept on the hotplate over night at 120 °C. Afterwards, the aqua regia was evaporated at 100°C. After cooling down the dry samples to 55 °C, 150 µl of 14 mol l^{-1} HNO_3 and 150 µl of 30 % H_2O_2 were added and evaporated. The aqua regia step and the subsequent H_2O_2-HNO_3 step were performed to destroy organic compounds. Then the dry samples were diluted and dissolved with adequate amounts of 3 % HNO_3 in closed screw-top Savillex beakers for 12 – 24 hours at 120°C. Adequate amounts of the diluted sample solutions, aiming for a total U amount of 400 – 500 ng, were weighed in 7 ml screw-top Savillex beakers and spiked with the ^{233}U-^{236}U double spike IRMM 3636-A with a spike sample ratio of ca. 0.03 (Weyer et al., 2008). In order to minimize peak tailing effects, the spike/sample mixtures for all samples and standards were adjusted to a $^{236}U/^{235}U$-ratio of approximately 3 (± 10 %). The amount of spike was calculated with the following equation:

$$m_{sp} = \frac{6 * m_{sa}}{c_{sp} * ab_{^{238}U/^{235}U}} \qquad (2)$$

m_{sp} ≙ spike weight
m_{sa} ≙ sample weight
ab ≙ natural abundance
c_{sp} ≙ spike concentration

Then the spike sample mix was evaporated over night at 100°C. Before the chemical separation of U from the remaining sample matrix, the dry sample was dissolved with 1 ml of 3 M HNO_3 for 12 – 24 hours. The chemical separation procedure with a chromatographic extraction method with Eichrom UTEVA was modified after Weyer et al. (2008) and Horwitz et al. (1992; 1993). The custom-made columns had a volume of 1 ml and an acid reservoir of 5 ml. After filling the columns with the UTEVA resin it was cleaned three times with 5 ml of 0.05 M HCl and then conditioned with 5 ml of 3 M HNO_3. In the next step the samples were loaded onto the UTEVA resin and rinsed once with 1 ml and thrice with 5 ml of 3 M HNO_3 to remove most matrix elements. Purification of U from Th was unnecessary as the synthetic samples of the mobilization experiments did not contain any Th. For that reason, the extraction protocol was continued with the conditioning with 5 ml of 7.5 M HCl. As the final step the U was collected in 15 ml screw-top Savillex beakers via eluting with thrice 1 ml, once each 3 ml

and 5 ml of 0.05 M HCl. The eluate was evaporated over night at 105 °C and each 100 µl of 14 M HNO_3 and 100 µl of 30 % H_2O_2 were added and evaporated afterwards. Then the dry samples were re-dissolved in 1 ml of 3 % HNO_3 for 12 to 24 hours at 120 °C in closed beakers. Finally, the adequate amounts of the sample solutions were diluted with adequate amounts of 3 % HNO_3 in order to receive 4 ml sample solution with about 70 ng g^{-1} U.

2.6. ICP-MS and MC-ICP-MS measurement

The concentration measurements were carried out with a Thermo Scientific Element XR HR-ICP-MS (inductively coupled plasma mass spectrometry) and the isotope measurements with a Thermo-Scientific Neptune MC-ICP-MS (multi collector ICP-MS), whereby all isotope variations were reported relative to the composition of the IRMM-184 standard. The isotope measurements were performed similar to the protocol published by (Noordmann et al., 2015). The Element XR was equipped with a secondary electron multiplier (SEM) and a faraday cup and an ESI SC2-DX autosampler as the sample introduction system. Additionally, a glass spray chamber, a PFA nebulizer with uptake rates of 100 µl min^{-1}, a solution injector with 1.8 mm in diameter and nickel (Ni) H-cones as skimmer and sample cone were used. Multi-element standard solutions were used for the external standardization of the concentration measurements. Argon was used as the sample gas. The measurements were performed in low resolution with an internal standardization with an iridium solution (Alfa-Aesar, Germany), which was added to all samples and standards (5 ng/g), 3 runs and 3 passes and a take up time of 70 s. With this protocol, a precision of 2SD (two standard deviation) < 5% was achieved. Between the measurements the system was rinsed for 6 min with 5 % HNO_3. Before the first measurement and after about 20 measurements, depending on the sample sets, a new calibration line with 0.5, 1, 5, 10 and 50 ppb U-samples was measured.
The Thermo-Scientific Neptune MC-ICP-MS was equipped with 10 faraday detectors, eight amplifiers with 10^{11} Ω, one amplifier with 10^{10} Ω (^{238}U) and one amplifier with 10^{13} Ω (^{234}U) and an ESI SC2-DX autosampler combined with a Cetac Aridus II as the sample introduction system. The temperature of the PFA spray chamber of the Cetac Aridus II was kept at 110 °C and the temperature of the PTFE membrane at 160 °C. The PFA nebulizer enabled uptake volumes of 100 µl min^{-1}. The MC-ICP-MS was configurated as follows: ^{232}Th (L3), ^{233}U (L2), ^{234}U (L1), ^{235}U (Center), ^{236}U (H1), ^{238}U (H3). Throughout the analysis, a standard Ni sampler cone and a Ni X skimmer cone with a 0.8 mm copper spacer in between were used. Argon functioned as sample gas. The instrument was tuned to a sensitivity of typically 80 V per 70 ng/g for ^{238}U and the measurements were performed in low resolution. Before each analysis session the abundance sensitivity was determined on a spike-free solution. The tail correction was negligible as the sensitivity was ≤0.1 ppm of the ^{238}U signal at mass 236. Each U analysis was conducted with ~ 4 min total integration time consisting of 60 cycles with a take up time of

80 s. Each sample was measured thrice and the results were arithmetically averaged. Between the measurements the system was rinsed for 4 min with 5 % HNO_3. The samples were measured via a modified standard-sample bracketing (two samples between the standard measurements) with the standard IRMM-184, as this standard was used as the U source for the mobilization experiments, to correct the drift in the instrumental mass bias. The ^{233}U-^{236}U double spike IRMM-3636A was used to correct for instrumental mass bias with the exponential law (Yang and E. Sturgeon, 2003). The discrimination value was calculated with the following formula:

$$\beta = \frac{\ln\left[\frac{[^{x}U/^{y}U]_N}{[^{x}U/^{y}U]_M}\right]}{\ln\left[\frac{M^{xU}}{M^{yU}}\right]} \qquad (3)$$

N ≙ true ratio
M ≙ measured ratio
M^{xU}, M^{yU} ≙ mass of the isotope x or y

The results of the isotope measurements were expressed in the delta notation relative to the U standard IRMM-184. The precision of the replicate analysis of each sample was 2SD ≤ 0.07 ‰. Before each of the three sample rounds and after the last round a control sample and a set of standards was measured in order to check the reproducibility and accuracy. Therefore, the IRMM-184 and the Reimep 18A standard were measured relative to the CRM 112A standard. The results agreed with those previously reported in literature within uncertainties (Noordmann et al., 2015; Weyer et al., 2008). Uranium isotope ratios are reported using the δ notation (in ‰)

Table S5 | U isotope fractionation ($\delta^{238}U$ [‰]) observed during all mobilization experiments of non-crystalline U(IV) by *At. ferrooxidans* of this study

Experiment	time [h]	$\delta^{238}U$ [‰]	2SD	absolute $\delta^{238}U$ [‰]	Experiment	time [h]	$\delta^{238}U$ [‰]	2SD	absolute $\delta^{238}U$ [‰]
**biotic**					**biotic**				
active/ untreated (1)	0	0.00	0.02		prewashed (1)	0	0.05	0.01	
	0.05	-0.40	0.03	-0.40		0.05	-0.07	0.03	-0.12
	0.66	-0.13	0.02	-0.13		0.66	0.07	0.01	0.02
	1.5	-0.02	0.03	-0.01		1.5	0.10	0.02	0.05
	3	0.03	0.01	0.03		3	0.09	0.05	0.04
	25.75					25.75	0.09	0.05	0.05
	67.5	0.06	0.07	0.06		67.5	0.02	0.02	-0.02
	164	0.03	0.03	0.03		164	0.02	0.07	-0.03
					prewashed (2)	0	0.04	0.04	
						0.05	-0.09	0.02	-0.13
						0.66	0.02	0.04	-0.02
						1.5	0.07	0.02	0.03
						3	0.08	0.03	-0.04
						25.75	0.06	0.03	0.02
						67.5	0.06	0.02	0.02
						164	0.04	0.05	0.00
inactivated/ with formate (1)	0	-0.01	0.04		prewashed + inactivated/ formate (1)	0	0.04	0.06	
	0.05	-0.36	0.05	-0.35		0.05	-0.06	0.02	-0.09
	0.66	-0.08	0.04	-0.08		0.66	0.04	0.03	0.00
	1.5					1.5	0.08	0.02	0.04
	3	0.02	0.02	0.03		3	0.09	0.03	0.05
	25.75	0.07	0.02	0.07		25.75	0.06	0.03	0.02
	67.5	0.06	0.02	0.06		67.5	0.04	0.04	0.00
	164	0.02	0.06	0.03		164	0.04	0.02	0.00
inactivated/ with formate (2)	0	0.01	0.03		prewashed + inactivated/ formate (2)	0	0.04	0.04	
	0.05	-0.32	0.02	-0-33		0.05	-0.04	0.04	-0.08
	0.66	-0.23	0.03	-0.23		0.66	0.06	0.01	0.02
	1.5	-0.14	0.03	-0.15		1.5	0.07	0.04	0.03
	3	-0.07	0.03	-0.08		3	0.09	0.02	0.05
	25.75	0.04	0.06	0.03		25.75	0.07	0.03	0.02
	67.5	0.06	0.03	0.05		67.5	0.04	0.03	0.00
	164	0.05	0.02	0.04		164	0.03	0.05	-0.02

Table S6 | Mobilized fraction (c/c_0) of non-crystalline U(IV) by *At. ferrooxidans* over time

Experiment	time [h]	concentration [µg/g]	c/c_0	Experiment	time [h]	concentration [µg/g]	c/c_0
**biotic**				**biotic**			
active/ untreated (1)	0	48.382	-	prewashed (1)	0	43.559	-
	0.05	1.898	0.04		0.05	10.020	0.23
	0.66	10.798	0.22		0.66	23.459	0.54
	1.5	18.274	0.38		1.5	30.796	0.71
	3	22.955	0.47		3	33.339	0.77
	19.75	31.006	0.64		19.75	36.407	0.84
	25.75	33.624	0.69		25.75	37.567	0.86
	43	36.593	0.76		43	38.533	0.88
	67.5	39.736	0.82		67.5	40.260	0.92
	91.75	41.935	0.87		91.75	40.962	0.94
	164	42.963	0.89		164	41.278	0.95
active/ untreated (2)	0	54.724	-	prewashed (2)	0	43.940	-
	0.05	1.055	0.02		0.05	9.013	0.21
	0.66	6.454	0.12		0.66	19.300	0.44
	1.5	10.528	0.19		1.5	25.004	0.57
	3	13.496	0.25		3	29.002	0.66
	19.75	22.912	0.42		19.75	34.731	0.79
	25.75	24.162	0.44		25.75	36.855	0.84
	43	20.138	0.37		43	38.242	0.87
	67.5	20.092	0.37		67.5	40.188	0.91
	91.75	21.446	0.39		91.75	42.409	0.97
	164	24.315	0.44		164	43.148	0.98
inactivated/ with formate (1)	0	50.616	-	prewashed + inactivated/ formate (1)	0	48.562	-
	0.05	1.353	0.03		0.05	9.923	0.20
	0.66	15.403	0.30		0.66	17.887	0.37
	1.5	17.921	0.35		1.5	30.353	0.63
	3	23.528	0.46		3	32.670	0.67
	19.75	32.399	0.64		19.75	37.945	0.78
	25.75	35.756	0.71		25.75	39.200	0.81
	43	39.423	0.78		43	41.863	0.86
	67.5	42.371	0.84		67.5	43.556	0.90
	91.75	44.312	0.88		91.75	44.034	0.91
	164	46.763	0.92		164	44.381	0.91
inactivated/ with formate (2)	0	50.853	-	prewashed + inactivated/ formate (2)	0	41.673	-
	0.05	1.833	0.04		0.05	*10.187*	0.24
	0.66	6.804	0.13		0.66	21.930	0.53
	1.5	10.859	0.21		1.5	27.371	0.66
	3	14.973	0.29		3	30.058	0.72
	19.75	26.188	0.51		19.75	29.002	0.70
	25.75	32.574	0.64		25.75	35.712	0.86
	43	36.875	0.73		43	37.378	0.90
	67.5	40.184	0.79		67.5	39.438	0.95
	91.75	41.736	0.82		91.75	1.494	0.04
	164	43.390	0.85		164	40.044	0.96

Table S7 | Mobilized fraction (c/c_0) of non-crystalline U(IV) by abiotic mobilization over time

Experiment	time [h]	concentration [µg/g]	c/c_0	Experiment	time [h]	concentration [µg/g]	c/c_0
**control sample (abiotic)** untreated	0	51.648	-	**control sample (abiotic)** prewashed	0	39.495	-
	0.05	0.119	0.00		0.05	6.695	0.17
	0.66	1.253	0.02		0.66	13.364	0.34
	1.5	1.768	0.03		1.5	17.730	0.45
	3	2.456	0.05		3	20.114	0.51
	19.75	1.602	0.03		19.75	9.612	0.24
	25.75	2.226	0.04		25.75	8.453	0.21
	43	1.057	0.02		43	6.530	0.17
	67.5	1.406	0.03		67.5	6.792	0.17
	91.75	1.933	0.04		91.75	7.414	0.19
	164	2.749	0.05		164	8.246	0.21
with formate (1)	0	52.396	-	prewashed + formate (1)	0	45.498	-
	0.05	0.136	0.00		0.05	6.295	0.14
	0.66	1.848	0.04		0.66	12.846	0.28
	1.5	3.311	0.06		1.5	18.575	0.41
	3	4.096	0.08		3	20.135	0.44
	19.75	3.006	0.06		19.75	8.348	0.18
	25.75	2.459	0.05		25.75	7.442	0.16
	43	1.507	0.03		43	5.540	0.12
	67.5	1.375	0.03		67.5	5.567	0.12
	91.75	1.380	0.03		91.75	5.938	0.13
	164	1.066	0.02		164	6.766	0.15
with formate (2)	0	62.314	-	prewashed + formate (2)	0	39.083	-
	0.05	0.095	0.00		0.05	6.919	0.18
	0.66	0.750	0.01		0.66	9.915	0.25
	1.5	0.614	0.01		1.5	11.805	0.30
	3	0.494	0.01		3	13.356	0.34
	19.75	0.209	0.00		19.75	10.976	0.28
	25.75	0.257	0.00		25.75	9.958	0.25
	43	0.141	0.00		43	5.888	0.15
	67.5	0.139	0.00		67.5	4.414	0.11
	91.75	0.142	0.00		91.75	4.258	0.11
	164	0.090	0.00		164	6.032	0.15

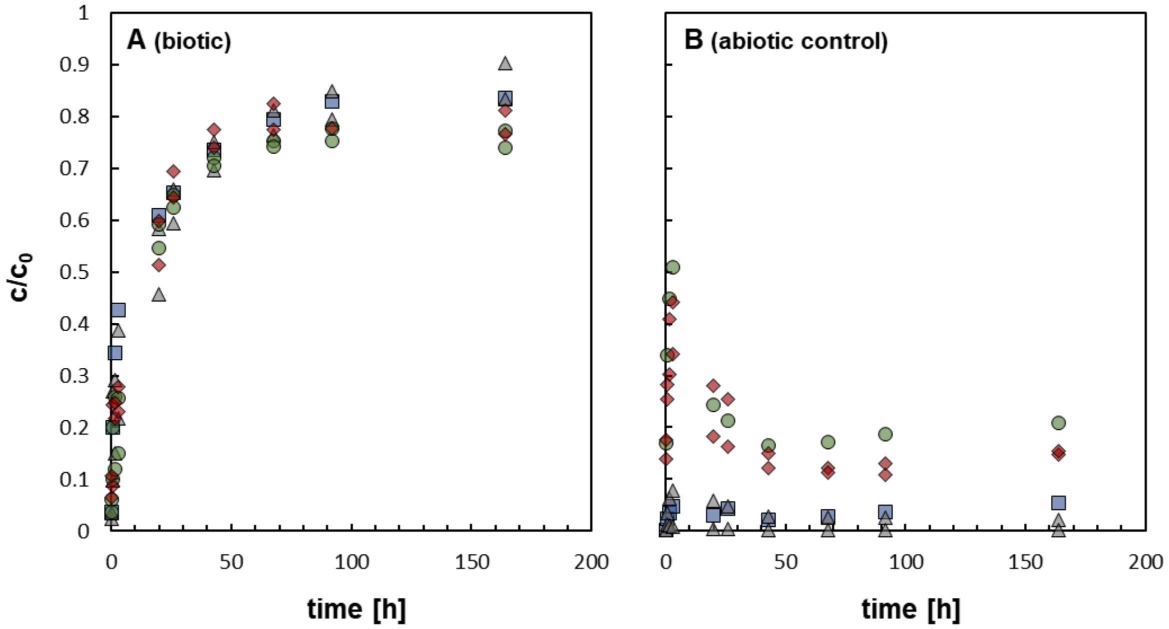

FIGURE S1| | (A) relative U concentration (c/c_0) of mobilized non-crystalline U(IV) by *At. ferrooxidans* against time; control-sample-corrected (respective data Table S6, SI)

(B) abiotically mobilized non-crystalline U(IV) displayed as relative U concentration (c/c_0) against time (respective data Table S7, SI)

The different experimental setups are distinguished as follows:

□ active/ untreated △ inactivated/ with formate ○ prewashed

◇ prewashed + inactivated/ formate

References

Beller, H. R. (2005). Anaerobic, Nitrate-Dependent Oxidation of U(IV) Oxide Minerals by the Chemolithoautotrophic Bacterium Thiobacillus denitrificans. *Appl. Environ. Microbiol.* 71, 2170–2174. doi: 10.1128/AEM.71.4.2170-2174.2005.

German Collection of Microorganisms and Cell Cultures GmbH: Thiobacillus denitrificans DSM 12475. Available at: https://www.dsmz.de/collection/catalogue/details/culture/DSM-12475 [Accessed February 6, 2021].

German Collection of Microorganisms and Cell Cultures GmbH: Acidithiobacillus ferrooxidans DSM 14882. Available at: https://www.dsmz.de/collection/catalogue/details/culture/DSM-14882 [Accessed February 6, 2021].

Horwitz, E. P., Chiarizia, R., Dietz, M. L., Diamond, H., and Nelson, D. M. (1993). Separation and preconcentration of actinides from acidic media by extraction chromatography. *Anal. Chim. Acta* 281, 361–372. doi: 10.1016/0003-2670(93)85194-O.

Horwitz, E. P., Dietz, M. L., Chiarizia, R., Diamond, H., Essling, A. M., and Graczyk, D. (1992). Separation and preconcentration of uranium from acidic media by extraction chromatography. *Anal. Chim. Acta* 266, 25–37. doi: 10.1016/0003-2670(92)85276-C.

Noordmann, J., Weyer, S., Montoya-Pino, C., Dellwig, O., Neubert, N., Eckert, S., et al. (2015). Uranium and molybdenum isotope systematics in modern euxinic basins: Case studies from the central Baltic Sea and the Kyllaren fjord (Norway). *Chem. Geol.* 396, 182–195. doi: 10.1016/j.chemgeo.2014.12.012.

Stylo, M., Alessi, D. S., Shao, P. P., Lezama-Pacheco, J. S., Bargar, J. R., and Bernier-Latmani, R. (2013). Biogeochemical Controls on the Product of Microbial U(VI) Reduction. *Environ. Sci. Technol.* 47, 12351–12358. doi: 10.1021/es402631w.

Wakeman, K., Auvinen, H., and Johnson, D. B. (2008). Microbiological and geochemical dynamics in simulated-heap leaching of a polymetallic sulfide ore. *Biotechnol. Bioeng.* 101, 739–750. doi: 10.1002/bit.21951.

Weyer, S., Anbar, A. D., Gerdes, A., Gordon, G. W., Algeo, T. J., and Boyle, E. A. (2008). Natural fractionation of 238U/235U. *Geochim. Cosmochim. Acta* 72, 345–359. doi: 10.1016/j.gca.2007.11.012.

Yang, L., and E. Sturgeon, R. (2003). Comparison of mass bias correction models for the examination of isotopic composition of mercury using sector field ICP-MS. *J. Anal. At. Spectrom.* 18, 1452–1457. doi: 10.1039/B307973B.

Zhang, R., Hedrich, S., Römer, F., Goldmann, D., and Schippers, A. (2020). Bioleaching of cobalt from Cu/Co-rich sulfidic mine tailings from the polymetallic Rammelsberg mine, Germany. *Hydrometallurgy* 197, 105443. doi: 10.1016/j.hydromet.2020.105443.
